# Exploring the association between sweet liking and treatment response to naltrexone in patients with alcohol use disorder

**DOI:** 10.1093/ijnp/pyag004

**Published:** 2026-01-23

**Authors:** Min-Chiao Chang, Chun-Hsin Chen, Shih-Chun Meng, Shu-Hao Hsu, Hu-Ming Chang, Ming-Chyi Huang

**Affiliations:** Department of Psychiatry, Mackay Memorial Hospital, 92, Section 2, Zhongshan North Road, Zhongshan District, Taipei, Taiwan; Department of Psychiatry, Wan-Fang Hospital, Taipei Medical University Hospital, 111, Section 3, Xinglong Road, Wenshan District, Taipei, Taiwan; Department of Psychiatry, School of Medicine, College of Medicine, Taipei Medical University, 250, Wuxing Street, Xinyi District, Taipei, Taiwan; Psychiatric Research Center, Wang-Fang Hospital, Taipei Medical University, 111, Section 3, Xinglong Road, Wenshan District, Taipei, Taiwan; Department of Addiction Sciences, Taipei City Psychiatric Center, Taipei City Hospital, 309, Song-de Road, Xinyi District, Taipei, Taiwan; Department of Addiction Sciences, Taipei City Psychiatric Center, Taipei City Hospital, 309, Song-de Road, Xinyi District, Taipei, Taiwan; Department of Addiction Sciences, Taipei City Psychiatric Center, Taipei City Hospital, 309, Song-de Road, Xinyi District, Taipei, Taiwan; Department of Psychiatry, School of Medicine, College of Medicine, Taipei Medical University, 250, Wuxing Street, Xinyi District, Taipei, Taiwan; Psychiatric Research Center, Wang-Fang Hospital, Taipei Medical University, 111, Section 3, Xinglong Road, Wenshan District, Taipei, Taiwan; Department of Addiction Sciences, Taipei City Psychiatric Center, Taipei City Hospital, 309, Song-de Road, Xinyi District, Taipei, Taiwan; Psychiatric Research Center, Taipei Medical University Hospital, Taipei Medical University, 250, Wuxing Street, Xinyi District, Taipei, Taiwan

**Keywords:** sweet-liking phenotype, alcohol use disorder, naltrexone, alcohol drinking outcomes

## Abstract

**Background:**

Response to naltrexone varies among individuals with alcohol use disorder (AUD). Studies in Western populations have linked the sweet-liking (SL) phenotype, reflecting an individual’s hedonic response to sweet taste, to improved outcomes with naltrexone. However, cultural and ethnic variations in SL raise questions about the generalizability of this association. This exploratory investigation examined the relationship between SL phenotype and naltrexone treatment outcomes in Taiwanese patients with AUD.

**Methods:**

Patients with DSM-5-defined severe AUD who had completed alcohol withdrawal were enrolled in an 8-week open-label naltrexone trial. Participants completed a sweet taste test and were categorized into SL or sweet-disliking (SDL) groups. Outcomes included alcohol consumption (drinking days per week, heavy drinking days per week, drinks per drinking day, abstinent days), alcohol craving, and depression and anxiety severity. Generalized estimating equations were used to assess the effects of time, group, and their interaction.

**Results:**

Ninety-one patients were enrolled (SL: *n* = 44; SDL: *n* = 47). Baseline characteristics were comparable between groups, except for smoking-related variables. After adjusting for smoking, both groups showed improvements in alcohol use and psychological measures over time. A significant group-by-time interaction was found for drinks per drinking day, with the SL group showing more favorable outcomes than the SDL group (*P* = .03).

**Conclusions:**

This is the first exploratory investigation in Asians to assess SL phenotype in relation to naltrexone response in AUD patients. The SL phenotype was associated with reduced alcohol consumption over 8 weeks of naltrexone treatment, supporting SL phenotype as a potential biomarker for personalized AUD treatment.

## Graphical Abstract

**Graphical Abstract:**
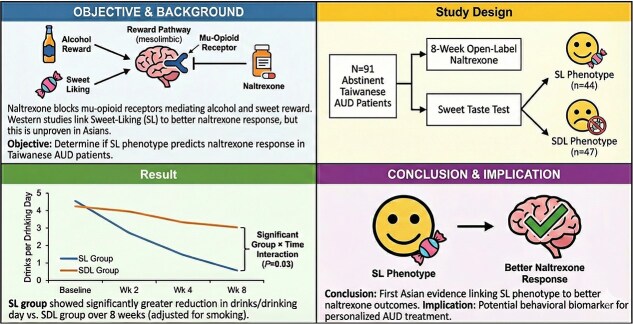

Significance Statement Alcohol use disorder (AUD) is prevalent worldwide and contributes to both individual and societal disease burden. Naltrexone is an approved pharmacological treatment for AUD; however, treatment response varies among individuals. Understanding the predictor of treatment response is crucial for precision medicine in AUD. Since both the hedonic response to sweet taste and the mechanism of naltrexone involve the mu-opioid reward system, previous studies have shown that individuals with AUD who prefer sweet taste respond better to naltrexone treatment.

In this explorative study, we identified sweet-liking (SL) and sweet-disliking (SDL) phenotypes among individuals with AUD. We found that patients in the SL group had significantly fewer number of drinks per drinking day than those in the SDL group. As the first study in an Asian population to examine the association between SL and naltrexone treatment, our findings support the potential of the SL phenotype as a predictor to naltrexone treatment response.

## INTRODUCTION

Alcohol is a widely consumed substance with addictive properties and easy accessibility, contributing to a broad range of adverse consequences for both individuals and society.^1^ Recent research has identified a significant association between alcohol consumption and 61 distinct diseases among East Asian populations.^2^ Alcohol use disorder (AUD)—characterized by compulsive heavy alcohol use and loss of control over alcohol intake despite adverse consequences,^3^ is one of the most prevalent mental health disorders worldwide and a major contributor to the global burden of morbidity and mortality.^4^^,^^5^ In a cross-sectional survey involving 2979 individuals diagnosed with AUD, 77% suffered from moderate-to-severe co-occurring psychiatric and/or somatic conditions.^6^ In Taiwan, a mortality analysis revealed that 45% of patients suffering from AUD died within a 7-year follow-up period.^7^ Given the extensive and profound negative impact of AUD, there is an urgent need for effective treatment interventions.

Three medications have been approved by the U.S. FDA to treat AUD, including disulfiram, naltrexone, and acamprosate.^8^ Among them, naltrexone, a non-selective opioid antagonist, reduces the euphoric effects of alcohol by competitively blocking the mu-opioid receptor in the central nervous system, particularly the mesolimbic pathway, thereby modulating the dopamine-mediated rewarding effects of alcohol.^9^ Although naltrexone has garnered empirical evidence supporting its efficacy in treating AUD,^10^ findings across studies have been inconsistent.^11-15^ In Taiwan, a double-blind, placebo-controlled trial found that while naltrexone significantly reduced alcohol craving, relapse rates did not differ between the naltrexone and placebo groups (*P* = .671).^16^ The mixed results regarding naltrexone’s efficacy among individuals with AUD indicates substantial inter-individual differences in the response to naltrexone.^15^^,^^17^ Consequently, identifying the distinct patient characteristics that influence therapeutic response to naltrexone is critical for developing more effective and personalized treatment strategy.^18^^,^^19^

The hedonic response to sweet taste is a universally observed trait in humans, which is associated with the activity of the endogenous opioid system, particularly the mu-opioid system^20^^,^^21^ that also plays a pivotal role in the rewarding effects of alcohol.^22^ While literature uses varying terminology such as sweet preference, the hedonic response to sweet-taste is referred to as sweet-liking (SL) phenotype in this study. This trait can be broadly categorized into two phenotypes: SL and sweet-disliking (SDL), representing individuals who prefer or dislike sweet taste, respectively.^23^ SL is considered a stable and heritable trait;^24^^,^^25^ however, its phenotypic expression can be modulated by environmental and physiological factors. Both animal models and human studies have linked SL with risk of excessive alcohol consumption and the development of AUD.^26^^,^^27^ In consistent, individuals with AUD have been found to exhibit a greater SL compared to those without AUD.^28^ However, the extent to which SL influences alcohol drinking behavior remains unclear.^29^ Naltrexone has been shown to be more effective in individuals with SL phenotype, which is considered a behavioral marker of opioid-mediated reinforcement.^30^^,^^31^ The SL phenotype may therefore serve as a predictive indicator of treatment response to naltrexone.^30^^,^^32^ Given that SL is influenced by cultural and ethnic factors^25^ with Asians generally displaying lower SL compared to Western cultures,^33^ it remains unclear whether these findings are generalized across populations. To the best of our knowledge, no studies have investigated the relationship between SL phenotypes and response to naltrexone treatment in Asian individuals.

Given the potential of SL phenotype as a behavioral marker of naltrexone responsiveness and the known cultural variations in taste preferences. This exploratory study aimed to investigate the relationship between SL phenotypes and treatment response to naltrexone in patients with AUD in Taiwan. We followed patients who received naltrexone treatment for 8 weeks following alcohol abstinence and subsequently compared alcohol drinking outcomes between those with SL phenotype and those with SDL phenotype. We hypothesized that, consistent with the findings in Western populations, patients with SL phenotype would exhibit a more favorable clinical response to naltrexone treatment. Confirming this association could offer a simple, non-invasive behavioral measure to help optimize treatment allocation for AUD across diverse populations.

## METHODS

### Study Settings and Participants

This 8-week study was conducted at the Department of Addiction Sciences, Song-De branch of Taipei City Hospital (also known as Taipei City Psychiatric Center) between January 2022 and September 2024. The study was carried out in accordance with the principles of the Declaration of Helsinki and the Good Clinical Practice Guidelines. Ethical approval was obtained from the Research Ethics Committee of Taipei City Hospital (TCHIRB-11004002). Patients underwent a comprehensive medical evaluation and a structured psychiatric interview based on the Diagnostic and Statistical Manual of Mental Disorders, Fifth Edition^34^ to determine eligibility. All eligible individuals received a detailed explanation of the study and were enrolled after providing written informed consent.

The inclusion criteria were as follows: (1) aged between 20 and 65 years; (2) fulfillment of DSM-5 criteria for severe AUD; (3) maintenance of complete abstinence from alcohol for at least 7 days without withdrawal symptoms; and (4) a negative urine toxicology screen for opiates, amphetamines, ketamine, and cannabis. The exclusion criteria were as follows: (1) a current DSM-5 diagnosis of any substance use disorders other than tobacco use disorder; (2) a history of opioid use disorder or regular opioid use; (3) clinically significant medical conditions, including but not limited to cardiovascular disease; (4) major psychiatric disorders, including depression with suicidal ideation, bipolar disorder, schizophrenia or schizoaffective disorder; (5) evidence of hepatic impairment, defined as elevated bilirubin levels, documented cirrhosis, or aspartate aminotransferase (AST)^35^ or alanine aminotransferase (ALT) levels exceeding three times the upper limit of normal^35^; (6) homelessness; and (7) cognitive impairment that would interfere with the ability to understand the informed consent or comply with study procedures.

### Study Procedures

We interviewed eligible patients to collect sociodemographic data and alcohol drinking history (eg, age at first drink, age meeting the diagnosis of severe AUD, duration of AUD). Alcohol consumption patterns before detoxication (baseline) were assessed using the Time-Line Follow-Back method to evaluate the frequency and quantity of drinking in the past one month. In this study, one standard drink was defined as containing 10 grams of pure alcohol. Heavy drinking was defined as alcohol consumption exceeding 6 drinks per day for males and 4 drinks per day for females. Baseline clinical and laboratory assessments were collected. The baseline laboratory assessments included AST, ALT, γ- glutamyltransferase (GGT), total bilirubin (Bil-T), and mean corpuscular volume (MCV) of red blood cell. The baseline clinical assessment included the followings:

The Severity of Alcohol Dependence Questionnaire (SADQ), which has been widely used for measuring alcohol dependence severity.^36^

Smoking history was assessed via self-reported smoking status (never vs. ever smoker), cumulative pack-years,^37^ and Fagerstrom Test for Nicotine Dependence (FNTD).^38^

Alcohol craving was assessed using both the Visual Analogue Scale (VAS) and the Penn Alcohol Craving Scale (PACS).^39^ The VAS is a self-rated measure employing a 10-point Likert scale ranging from 0 to 9, where a score of 0 indicates no craving and a score of 9 signifies an intensity of craving so severe that the individual would be unable to resist a drink if it were available. The PACS is a five-item self-rated questionnaire that evaluates the frequency, intensity, and duration of alcohol craving, as well as the individual’s perceived ability to resist drinking.

Symptoms of depression and anxiety were measured using the Chinese versions of the 21-item Beck Depression Inventory (BDI)^40^ and the 21-item Beck Anxiety Inventory,^41^^,^^42^ respectively.

Following an initial assessment and 7-14 days of alcohol abstinence to ensure the resolution of alcohol withdrawal symptoms, which is defined as CIWA-Ar scores below 8 sustained over 72 hours without the need for additional medication. Then naltrexone hydrochloride was initiated at 25 mg/day for 14 days, then titrated to 50 mg/day for the remaining 6 weeks of treatment period. All participants received medical follow-up on an outpatient basis.

### Sweet Taste Test

The sweet taste test was administered between week 2 and week 4 of naltrexone use to evaluate participants’ sensitivity and hedonic response toward sweet taste, following procedures described by Iatridi, et al.^43^ Participants tasted the sucrose solutions and rated both perceived intensity and pleasurableness. Six concentrations of sucrose solution (0.05, 0.1, 0.2, 0.4, 0.6, and 0.8 M), each in 15 c.c, were presented in a pseudorandom order in five separated rounds, resulting in a total of 30 tastings. For each tasting, participants were instructed to sip the solution, swish it around their mouth, sip it out, and then rinse their mouth with distilled water. They were then asked to rate the intensity of sweetness by responding to the question, “How sweet is the taste?” Using a 100-mm VAS, anchored from 0 (“not sweet at all”) to 100 (“extremely sweet”). Pleasurableness was assessed after each tasting by asking “How much do you like the taste?” Responses were recorded on a 200-mm VAS ranging from -100 (“dislike extremely”) to +100 (“like extremely”).

As shown in Supplementary Table S1, participants demonstrated the ability to discriminate between different glucose concentrations, as evidenced by higher sweetness intensity ratings with increasing glucose concentration. The average pleasurableness rating for each concentration across five rounds is also shown in Supplementary Table S1. Notably, the 0.05 M and 0.1 M glucose concentration have the higher average pleasurableness ratings, indicating these were the most pleasurable concentration among the patients. In each round, the top-rated concentration, defined as the concentration receiving the highest rating from the patients, was documented. Overall, 42.9% to 49.4% of participants rated the 0.05 M and 0.1 M concentrations as the top-rated ones in each round (Supplementary Table S1). Subsequently, participants were categorized as having a SL phenotype or a SDL phenotype based on their pleasurableness ratings of the 0.2 M glucose solution. This concentration was selected as a reference point because it represents a moderate level of sweetness, sufficient to elicit pleasurableness ratings variation without inducing ceiling or floor effects. The threshold for classification was determined by calculating the overall median of participants’ mean pleasurableness ratings across the five trials of the 0.2 M solution. Individuals with a mean rating of 0.2 M solution above this overall median of 0.2 M solution were categorized as having a SL phenotype (48.8% of entire sample), while those with lower scores were classified as having a SDL phenotype (51.6% of entire sample). In addition, we graphically presented both the intensity and pleasurableness curves across concentration between the SL and the SDL groups. Supplementary Figure S1 presents that intensity ratings increased linearly with concentrations for all participants and were comparable between the two groups, indicating intact and similar sensory perception. Supplementary Figure S2 illustrates distinct sweet liking response for the SL and SDL groups across all tested concentrations. Taken together, these observations demonstrated that the phenotypic difference between group is specific to sweet liking, rather than sensory perception.

### Outcome Measurement

Four primary indices of alcohol use were re-assessed at week 2, week 4, and week 8: (1) number of drinking days per week, defined as days with any alcohol consumption; (2) number of heavy drinking days per week; (3) number of drinks per drinking days; and (4) number of abstinence days. We recorded number of drinking days and abstinence days as distinct outcomes. Drinking days quantify the frequency of pathological behavior and facilitate comparisons in pharmacotherapy trials,^44^ whereas abstinent days reflect early neurobiological recovery associated with normalization of prefrontal-striatal circuits, which predicts better long-term outcomes.^45^ In addition, VAS, PACS, BDI, and BAI scores were re-assessed at week 4 and week 8.

### Statistical Analysis

We used Chi-square tests for categorical variables and Mann-Whiney U tests for continuous variables, respectively, to examine the demographic differences between the SL and SDL groups. Generalized Estimation Equation (GEE) model was used to evaluate the naltrexone treatment response on alcohol drinking and psychological outcomes between the SL and SDL groups during 8-week intervention. Missing data were addressed using multiple imputation, generating five imputed datasets (m = 5), before conducting the GEE analyses. *P*-values from the GEE models were then adjusted using the false discovery rate (FDR) procedure to control for multiple testing. Statistical analysis was performed by IBM SPSS Statistics (Version 26).

## RESULTS

### Demographic, Clinical Characteristics

A total of 91 patients were enrolled in the study, with a mean age was 45.6 years and 72.5% were male. Among them, 44 participants were classified into the SL group and 47 into the SDL group. Table 1 shows the demographic and clinical characteristics and biochemical data between the SL and SDL groups. No significant differences in demographic and clinical characteristics and biochemical data were observed between the SL and SDL groups, except that participants in the SDL group were significantly more likely to be smokers (78.7% vs. 51.1%, *P* = .04) and had a higher cumulative tobacco exposure, as indicated by greater pack-years (22.8 ± 20.8 vs. 14.4 ± 20.5, *P* = .02), compared to those in the SL group.

**Table 1:** Baseline sociodemographic, clinical, and laboratory characteristics of the SL and SDL Groups.

	**SL group (*n* = 44)**	**SDL group (*n* = 47)**	***P***
**Sociodemographic**			
**Age, year**	44.8 ± 10	46.3 ± 16.8	.61
**Male**	30 (63.8)	36 (76.6)	.48
**BMI, kg/m^2^**	22.3 ± 4	22.6 ± 3.8	.53
**Education, year**	14 ± 3.5	13.4 ± 5.5	.42
**Smoker**	24 (51.1)	37 (78.7)	**.04**
**Pack-years of smoking**	14.4 ± 20.5	22.8 ± 20.8	**.02**
**FTND**	3.1 ± 3.5	4.7 ± 3.6	.06
**Drinking history**			
**Age at first alcohol use, year**	19.7 ± 8.5	19.4 ± 8	.86
**Age of onset of severe AUD, year**	32.7 ± 10.2	32.4 ± 13.7	.79
**Duration of AUD, year**	25.1 ± 11.7	26.8 ± 13.2	.72
**Psychological characteristics at baseline**			
**SADQ score**	18.4 ± 10.3	18.3 ± 13	.6
**VAS score**	64.5 ± 26.7	59.5 ± 33.2	.91
**PACS score**	17.2 ± 7.1	17.3 ± 8.5	.62
**BDI score**	20.8 ± 10	18.8 ± 13.9	.69
**BAI score**	14.1 ± 11.3	11.3 ± 11.2	
**Laboratory characteristics at baseline**			
**AST, U/L**	52.1 ± 83.3 (*n* = 41)	41 ± 83.4 (*n* = 48)	.25
**ALT, U/L**	45.6 ± 36.8 (*n* = 37)	29 ± 36.9 (*n* = 45)	.3
**GGT, U/L**	234.5 ± 262 (*n* = 31)	112.4 ± 262 (*n* = 30)	.82
**Bil-T, mg/dL**	0.7 ± 1.1 (*n* = 17)	1.4 ± 1.1 (*n* = 26)	.56
**MCV, fL**	93.5 ± 62.4 (*n* = 34)	92.2 ± 62.6 (*n* = 40)	.31

Data are presented with mean ± SD or n (%).

Chi-square test was used for categorical variable and Mann–Whitney U tests. *P-*values in bold indicate those <.05.

Abbreviations: ALT, alanine aminotransferase; AST, aspartate aminotransferase; AUD, alcohol use disorder; BAI, Beck Anxiety Inventory; BDI, Beck Depression Inventory; Bil-T, total bilirubin; BMI, body mass index; FTND, Fagerstrom Test for Nicotine Dependence; GGT, gamma-glutamyl transpeptidase; MCV, mean corpuscular volume; PACS, Penn Alcohol Craving Score; SADQ, Severity of Alcohol Dependence Questionnaire; SD, standard deviation; SDL, sweet-disliking; SL, sweet-liking; VAS, Visual Analog Score for craving.

### Alcohol Drinking Outcomes

Table 2 shows the alcohol drinking outcomes following naltrexone treatment between the SL and SDL groups. Both groups demonstrated significant improvements in all alcohol drinking outcomes over time. A significant group $\times$ time interaction effect was observed for the number of drinks per drinking day, while the main effect of group was not statistically significant. This suggests that over time, participants in the SL group demonstrated significantly a greater reduction in the number of drinks per drinking day compared to those in the SDL groups. Supplementary Figure S3 visualizes the changes in the number of drinks per drinking day for both groups throughout the 8-week treatment. While both groups showed a reduction, the curves diverged over time, with the SL group exhibiting a greater reduction.

**Table 2:** Alcohol drinking outcomes following naltrexone treatment over time between the SL and SDL groups.

	**Week0**	**Week2**	**Week4**	**Week8**	***P***		
**Number of patients, *n***					**Group effect**	**Time effect**	**Interaction effect**
**SL group**	44	43	40	37			
**SDL group**	47	47	47	35			
**Number of drinking days per week**					.15	**< .01**	.46
**SL group**	6.5 ± 1.2	1.3 ± 2.1	1.0 ± 1.6	0.9 ± 1.6			
**SDL group**	6.5 ± 2.3	1.7 ± 2	1.4 ± 1.7	1.6 ± 1.9			
**Number of heavy drinking days per week**					.28	**< .01**	.63
**SL group**	6.4 ± 1.5	0.6 ± 1.3	0.4 ± 0.9	0.5 ± 1.0			
**SDL group**	6.2 ± 2.6	0.7 ± 1.6	0.9 ± 1.4	1.0 ± 1.4			
**Number of drinks per drinking day**					.26	**< .01**	**.03**
**SL group**	14.8 ± 9.8	1.9 ± 3.2	1.2 ± 2.4	2.5 ± 3.6			
**SDL group**	12.8 ± 11.5	3.0 ± 7.0	4.0 ± 5.0	4.7 ± 4.7			
**Number of abstinence days**					.15	**< .01**	.46
**SL group**	0.5 ± 1.2	5.7 ± 2.1	6 ± 1.7	6.1 ± 1.9			
**SDL group**	0.5 ± 1.3	5.3 ± 2.4	5.6 ± 2.3	5.4 ± 2.8			

Data are presented with mean ± SD.

FDR-adjusted *P-values* were derived from GEEs (with 5 multiple imputations). *P-values* in bold indicate those <.05.

Abbreviations: SD, standard deviation; SDL, sweet-disliking; SL, sweet-liking.

### Psychological Outcomes

Table 3 shows changes of psychological outcomes following naltrexone treatment groups. Both SL and SDL groups showed significant psychological improvements over time; however, no significant group x time interaction effect was observed.

**Table 3:** Psychological outcomes following naltrexone treatment over time between the SL and SDL groups.

	**Week0**		**Week4**		**Week8**			***P***	
**Number of patients, n**							**Group effect**	**Time effect**	**Interaction effect**
**SL group**	44		40		37				
**SDL group**	47		47		35				
**VAS score**							.30	**< .01**	.64
**SL group**	64.5 ± 26.7		19.3 ± 24		18.8 ± 21.6				
**SDL group**	59.5 ± 33.2		21.9 ± 24.4		21.7 ± 25.5				
**PACS score**							.88	**< .01**	.97
**SL group**	17.2 ± 7.1		6.6 ± 6.7		6.3 ± 6.0				
**SDL group**	17.3 ± 8.5		6.4 ± 6.3		6.7 ± 7.1				
**BDI score**							.49	**< .01**	.64
**SL group**	20.8 ± 10.0		13.0 ± 11.7		14.2 ± 13.7				
**SDL group**	18.8 ± 13.9		12.5 ± 11.7		11.3 ± 11.9				
**BAI score**							.42	**< .01**	.87
**SL group**	14.1 ± 11.3		10 ± 10.2		9.4 ± 10.3				
**SDL group**	11.3 ± 11.2		7.9 ± 8.1		7.6 ± 7.5				

Data are presented with mean ± SD.

FDR-adjusted *P-values* were derived from GEEs was used in the analysis. *P-values* in bold indicate those <.05.

Abbreviations: BAI, Becker Anxiety Inventory; BDI, Becker Depression Inventory; PACS, Penn Alcohol Craving Scale; SD, standard deviation; SDL, sweet-disliking; SL, sweet-liking; VAS, Visual Analogue Scale.

## DISCUSSION

To our knowledge, this is the first study to examine the relationship between SL phenotype and response to naltrexone treatment in an Asian population. Consistent with the existing literature,^31^^,^^46^^,^^47^ our results demonstrated that patients receiving naltrexone treatment, either in the SL or SDL groups, exhibited improvements in drinking behaviors and psychological outcomes over an 8-week treatment period. Notably, patients in the SL group exhibited a greater reduction in the number of drinks per drinking day, suggesting that individuals with SL phenotype may experience more favorable treatment outcomes with naltrexone. These results imply that the therapeutic efficacy of naltrexone may be moderated by individual differences in SL.

Our findings are consistent with prior research indicating an association between SL phenotype and more favorable treatment responses to naltrexone.^30-32^ This association may be explained by the hedonic response to sweetness and its link to the endogenous opioid system,^48^ which is the pharmacological target of naltrexone. However, the specific alcohol drinking outcomes influenced by SL phenotypes vary across studies. In our study, the SL phenotype was associated with a greater reduction in the number of drinks per drinking days. However, previous research has linked SL to a reduced risk of relapse to heavy drinking^30^^,^^31^ or an increased number of abstinent days.^30^^,^^32^ Garbutt, et al.^32^ reported that the SL phenotype predicted favorable treatment outcomes only when considered in interaction between craving severity, suggesting that craving may be as an important moderator of in the relationship between the SL phenotype and naltrexone response. These findings suggest that SL phenotype could serve as a phenotypic marker for identifying individuals who may benefit more from opioid antagonist treatment, while also highlighting the complexity of behavioral and neurobiological interactions that influence therapeutic outcomes.

Our findings should be interpreted with caution with respect to the timing of the sweet taste test. Both abstinence duration and naltrexone exposure are known to affect SL, but in opposite directions. Abstinence has been associated with increased SL,^49^ while naltrexone has been shown to reduce SL through by blocking the opioid systems.^50^^,^^51^ In our study, sweet taste test was performed between 2 and 4 weeks after initiating naltrexone treatment, and following at least 7 days of abstinence. Consequently, the observed phenotypes may be effect by these two opposing factors. Prior studies differed in the abstinence requirements and the timing of sweet taste assessment. Garbutt, et al.^30^ encouraged but did not require abstinence and conducted the sweet taste test before starting naltrexone. Another study^32^ required only three days of abstinence and also performed the test before treatment initiation. Laaksonen, et al.^31^ did not specify an abstinence requirement and measured SL phenotype only after completion of a 32-week clinical trial. These methodological differences likely contributed to the variability in SL measurements and may affect the observed associations with treatment outcomes. Despite these methodological variations, all studies, including ours, consistently suggest that SL phenotype may affect naltrexone treatment response. However, the specific contributions of abstinence versus naltrexone exposure remain difficult to disentangle in the absence of serial measurements.

A major challenge in studying the relationship between SL phenotype and naltrexone treatment response is the lack of a universally accepted method for classifying SL and SDL phenotypes. Kampov-Polevoy, et al.^26^ defined SL individuals as those who rated the highest pleasantness for the most concentrated sucrose solution (0.83 M), which is a criterion widely adopted in subsequent studies. However, in our Taiwanese sample, no participants rated the 0.83 M solution as the most pleasant. Instead, the highest ratings were most frequently given to the 0.1 M and 0.05 M concentrations. Applying the 0.83 M criterion would therefore have yielded too few SL cases for meaningful analysis. Consequently, we adopted a modified approach, categorizing participants based on whether their average pleasantness rating of the 0.2 M sucrose solution across five trials was above or below the sample mean. The observed ethnic variation in SL likely reflects multifactorial influences, including genetic, environmental, and cultural factors. Genetic factors playing a critical role in shaping individual differences^52^ of sweet liking. Specifically, variants in the FTO gene and sweet-taste receptor genes TAS1R2 and TAS1R3 have been identified as contributors to individual variability in sweet perception. Hwang, et al.^53^ suggested that functional differences in TAS1R2 and TAS1R3 variants exist across ethnic groups, potentially explaining the lower SL observed in the Taiwanese sample compared to Western populations. A prior study found that Malaysian participants exhibited significantly lower overall liking for sweet foods compared to Caucasian Australians, despite preferring similar sucrose concentrations. This supports the general observation that Asians may have a lower sweet liking than Western populations.^54^ Environmental and cultural factors, such as early-life exposure to sweet foods^55^ and higher energy demands due to physically active lifestyles,^56^ also shape SL phenotype. Although early hedonic response to sweet are influenced by family and culture, they may shift with changing social contexts in adulthood.^57^ Lower SL in the Taiwanese cohort may attribute to TAS1R genetic variants, yet this predisposition interacts with environmental factors like dietary culture to shape the SL/SDL phenotype.

Our study revealed that participants in the SDL group were significantly more likely to be smokers and had greater cumulative smoking exposure. This reduced sweet liking may be partly explained by impaired sucrose sensitivity, as previous research linked smoking to diminished sweet taste perception.^58^ Additionally, chronic tobacco use has been associated with increased activation or altered regulation of mu-opioids receptors,^59^ which may attenuate the therapeutic efficacy of naltrexone—an opioid receptor antagonist. This aligns with prior findings indicating that individuals with co-occurring AUD and smoking history tend to respond less favorably to naltrexone.^60^ Given that smoking has been identified as a factor associated with worse naltrexone treatment outcomes,^61^ we statistically adjusted for smoking status to minimize its potential confounding effects. Importantly, even after controlling for smoking, the SDL group continued to exhibit reduced therapeutic responses (Table 2 and Table 3), supporting our hypothesis that SL phenotype may independently influences the efficacy of naltrexone in individuals with AUD.

It is crucial to reconcile the concept of SL as a stable trait with the observed variability due to smoking, naltrexone, and abstinence. We proposed that while genetic establish an individuals’ baseline trait, physiological states, and environmental and cultural factor act as modulators that shape the SL phenotype we observed. As mentioned, physiological states include chronic smoking, alcohol abstinence or naltrexone treatment itself. The SL phenotype measured in this study represents the net result of the stable genetic trait interacting with these dynamic physiological modulators.

Several limitations should be considered when interpreting our findings. First, the use of self-reported questionnaires may have introduced potential recall bias, potentially affecting the accuracy of clinical assessments over the study period. Second, a critical limitation concerns the timing of the sweet taste test. As mentioned above, because it was administered after both naltrexone initiation and alcohol abstinence, the SL phenotype was assessed under the combined influence of opioid blockade and alcohol abstinence. Future research should standardize abstinence duration and conduct the sweet taste test prior to naltrexone initiation. In addition, conducting the test at multiple time points (eg, after abstinence and before and during treatment) would help to distinguish stable genetic traits from medication effects. Third, the study was conducted within the addiction department of a psychiatric hospital, where participants generally presented with more severe AUD and were actively seeking treatment. This may limit the generalizability of the results to broader or less clinically severe populations. Fourth, the small sample size limited statistical power, and the possibility of residual false positives cannot be entirely excluded. Although a FDR correction was applied to reduce the risk of Type I error due to multiple comparisons, consistent trends were observed. A priori power analysis indicated that a sample size of 128 was required to detect a medium effect size (Cohen’s d = 0.5) with power 0.8 at a two-tailed α of 0.05. Therefore, the findings should be interpreted with caution and validated through replication in larger and more diverse cohorts. Finally, our study followed participants for only 8 weeks. Whether the observed effects persist over a longer treatment duration remains to be determined and warrants further investigation.

In conclusion, this exploratory study supports the notion that patients with severe AUD who exhibit a SL phenotype show more favorable responses to naltrexone treatment within a Taiwanese cohort, thereby extending the relevance of this association across diverse ethnic and cultural contexts. Future studies should clarify the neurobiological and genetic mechanisms linking the SL phenotype to naltrexone efficacy. Larger and more diverse samples, along with standardized sweet-taste testing protocols before naltrexone initiation, are needed to improve replicability and generalizability.

## Supplementary Material

Supplementary_Material_20251229_pyag004

## Data Availability

The data of this study are available on request from the corresponding author. The data are not publicly available because of privacy or ethical restrictions.
