# Correction to: Exploring the association between sweet liking and treatment response to naltrexone in patients with alcohol use disorder

**DOI:** 10.1093/ijnp/pyag017

**Published:** 2026-04-14

This is a correction to: Min-Chiao Chang, Chun-Hsin Chen, Shih-Chun Meng, Shu-Hao Hsu, Hu-Ming Chang, Ming-Chyi Huang, Exploring the association between sweet liking and treatment response to naltrexone in patients with alcohol use disorder, *International Journal of Neuropsychopharmacology*, Volume 29, Issue 2, February 2026, pyag004, https://doi.org/10.1093/ijnp/pyag004

The name of the fourth co-author has been emended to read: “Shu-Hao Hsu”.

