# Supplementary material for: Exploring the association between sweet liking and treatment response to naltrexone in patients with alcohol use disorder
Source: Int J Neuropsychopharmacol. 2026 Jan 23;29(2):pyag004. doi: 10.1093/ijnp/pyag004 (PMC12931563; doi:10.1093/ijnp/pyag004)
Supplement: Supplementary_Material_20251229_pyag004 [file supplementary_material_20251229_pyag004.docx]

| **Supplementary Table 1. Mean Ratings of Sweet Taste Intensity, Pleasurableness and Top-Rated Concentration by Round** | |  |  |
| --- | --- | --- | --- |
| ***n = 91*** | | | |
| **Average Pleasurableness Ratings for Each Concentration Across Five Rounds** |  |  |  |
| **Solution, M** | **Pleasurableness Rating** |  |  |
| 0.05 | 8.4 ± 53.6 |  |  |
| 0.1 | 9.3 ± 48.2 |  |  |
| 0.2 | 2.1 ± 39 |  |  |
| 0.4 | -20.4 ± 38.3 |  |  |
| 0.6 | -33.9 ± 44.4 |  |  |
| 0.8 | -40.5 ± 45.5 |  |  |
| **Average Intensity Ratings for Each Concentration Across Five Rounds** |  |  |  |
| **Solution, M** | **Intensity Rating** |  |  |
| 0.05 | 14.8 ± 15 |  |  |
| 0.1 | 20.7 ± 16.3 |  |  |
| 0.2 | 33.5 ± 19.4 |  |  |
| 0.4 | 60.5 ± 17.8 |  |  |
| 0.6 | 72.5 ± 17.5 |  |  |
| 0.8 | 80.2 ± 15.2 |  |  |
| **Top-Rated Concentration and the Pleasurableness Rating of Top-rated Concentration by Round** |  |  |  |
| **Round** | **Top-Rated Concentration, M** | **Number (%) of patients rating the concentration as top-rated** | **Pleasurableness Rating** |
| Round 1 | 0.1 | 45 (49.4%) | 12.3 ± 52.7 |
| Round 2 | 0.05 | 39 (42.9%) | 11 ± 60.9 |
| Round 3 | 0.1 | 42 (46.2%) | 19 ± 65.7 |
| Round 4 | 0.1 | 39 (42.9%) | 13.5 ± 62 |
| Round 5 | 0.05 | 39 (42.9%) | 23.2 ± 67.8 |
| Pleasurableness and intensity rating data are presented with mean ± SD. Abbreviations: M, molarity; SD, standard deviation. Top-rated concentration is based on the highest average pleasurableness rating per round. | | | |

**Supplementary Figure 1.** Intensity Ratings of Sucrose Solutions across Concentrations in the SL and SDL groups.


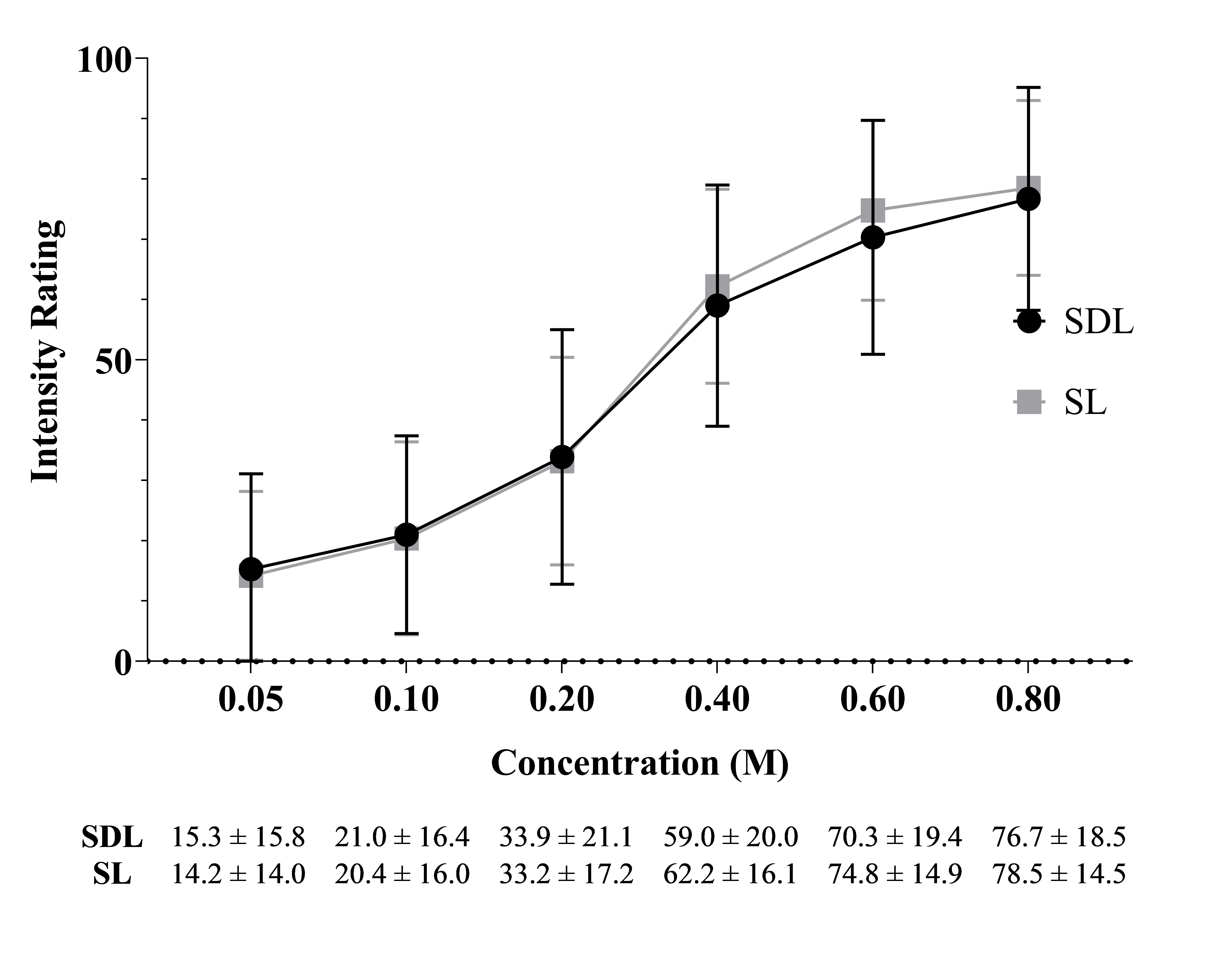


The graph illustrates the mean intensity ratings (± SD) for the SL group (grey squares) and the SDL group (black circles) across six sucrose concentrations. Data are presented as mean ± SD. Abbreviations: M, Molarity; SD, standard deviation, SDL, sweet-disliking; SL, sweet-liking.

**Supplementary Figure 2.** Intensity Ratings of Sucrose Solutions across Concentrations in the SL and SDL groups.


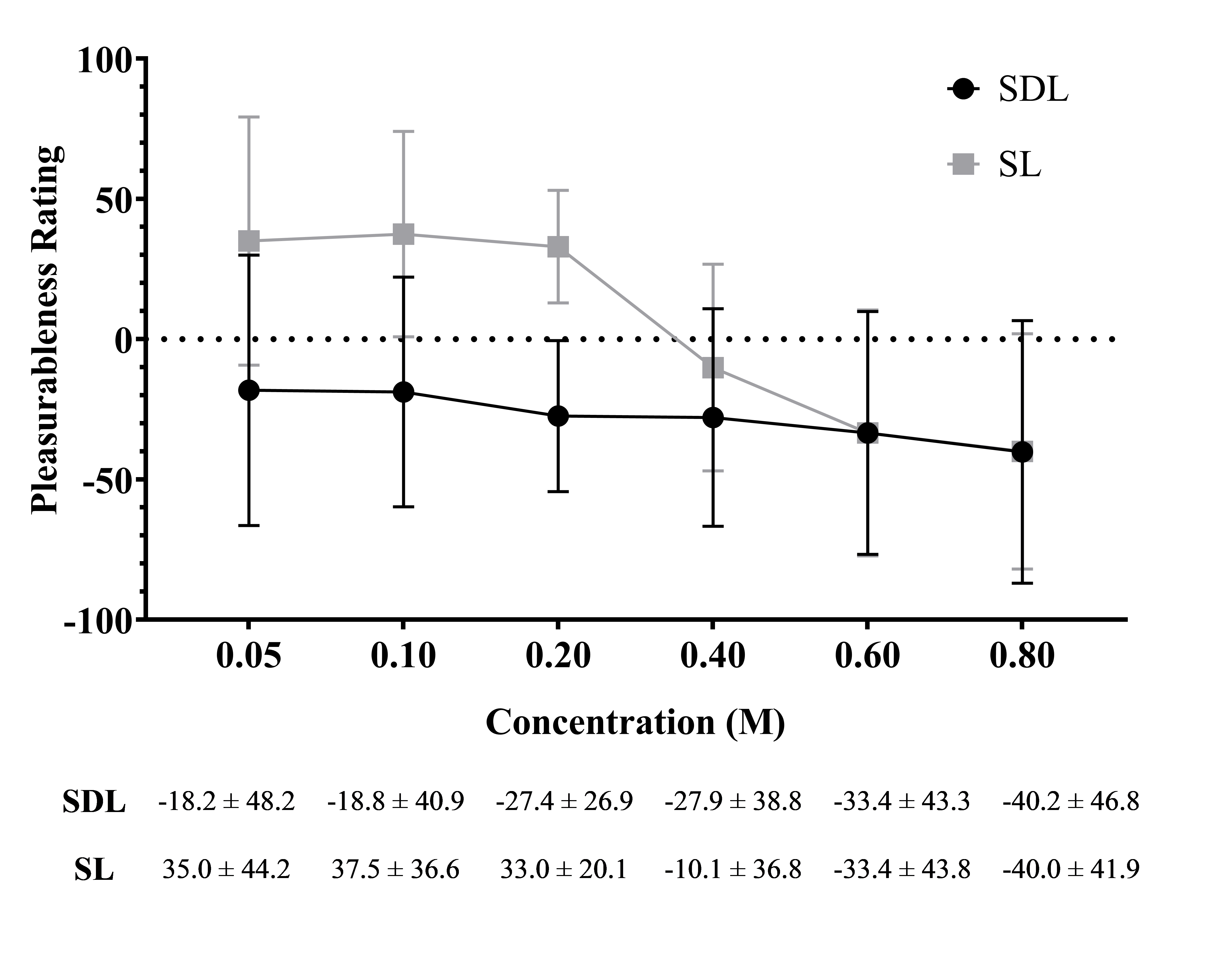


The graph illustrates the mean intensity ratings (± SD) for the SL group (grey squares) and the SDL group (black circles) across six sucrose concentrations. Data are presented as mean ± SD. Abbreviations: M, Molarity; SD, standard deviation, SDL, sweet-disliking; SL, sweet-liking.

**Supplementary Figure 3.** Changes in the Number of Drinks per Drinking Day in the SL and SDL groups over 8 weeks.


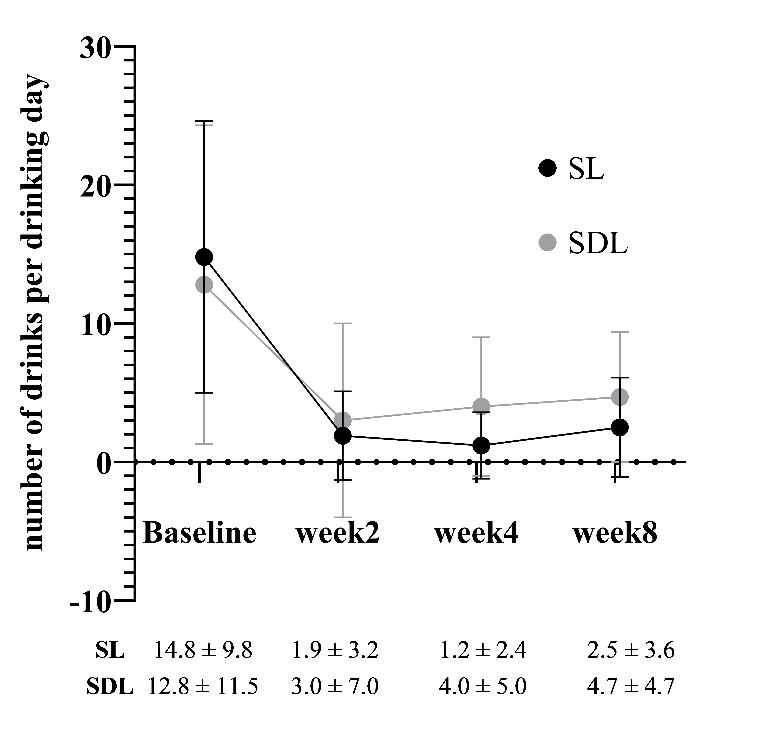


The mean number of drinks per drinking day was measured for the SL (black circles) and SDL (grey circles) groups at baseline, week 2, week 4, and week 8. Data are analyzed using a generalized estimating equation model. There was a significant main effect of time (*P* < 0.01) and a significant group × time interaction (*P*= 0.03). Data are expressed as mean ± SD. Abbreviations: SD, standard deviation, SDL, sweet-disliking; SL, sweet-liking.
